# Investigation of the Kinetics of Hysteresis Effects in Silica Gel

**DOI:** 10.3390/ma15176031

**Published:** 2022-09-01

**Authors:** Alexander Pöllmann, Matthias Reinelt, Heiko Briesen

**Affiliations:** 1 Process Systems Engineering, TUM School of Life Science Weihenstephan, Technical University of Munich, Gregor-Mendel-Str. 4, 85354 Freising, Germany; 2 Fraunhofer Institute for Process Engineering and Packaging IVV, Giggenhauser Str. 35, 85354 Freising, Germany

**Keywords:** sorption, kinetics, hysteresis, water, silica, model

## Abstract

Mathematical models can provide estimates for the shelf life of water-sensitive products like food or pharmaceuticals. This study presents a simple kinetic model using two first-order reactions for the evaporation and condensation of water. Furthermore, the model can be simplified to contain only one free parameter, the reaction rate constant *k*, which has been validated for silica gel at a relative humidity between 0% and 80% with experimental data. The experimental data shows the hysteresis effect of the silica gel in the region between 30% and 80% relative humidity and its dependence on the relative humidity earlier in the process. It also shows there are multiple equilibrium water contents at a relative humidity of 40%, depending on the previous relative humidity. The relative humidity barely influences the fitted reaction rate constant during adsorption. However, during the desorption process, not only the current relative humidity but also the history of relative humidity have an influence. A higher relative humidity in the previous step can slow down the desorption rate in the following step.

## 1. Introduction

Moisture content in the product and the headspace composition strongly influence the shelf life and quality of a wide variety of products, like food, pharmaceuticals, or even artworks [1,2,3]. Depending on the product, various conditions have to be fulfilled for extended shelf life. Pharmaceuticals need dry storage conditions below a specific relative humidity threshold to maintain their effectiveness [3,4]. Water-sensitive foods like mushrooms have to be stored at controlled relative humidity and water content to avoid drying at low relative humidity and bacterial spoilage at high relative humidity [2,5]. Due to these varying product demands, the optimal packaging solution can be unique for different products. A useful tool for packaging development is shelf-life modelling, which is the development of mathematical equations describing the different processes in the packaging (e.g., permeation or scavenging reactions) and the product (e.g., water sorption or oxidation) [6,7]. A shelf-life model can be employed to test different product/packaging combinations with minimal time-consuming storage tests [8]. This paper describes one of these fundamental processes: water adsorption and desorption on a solid. Mathematically, these processes could be described by the steady-state characteristics, i.e., a sorption isotherm, and the respective kinetic behaviour. The sorption isotherm describes the relationship between the water content in a solid at equilibrium and the relative humidity in the surrounding headspace and is different for every solid [8]. Langmuir used statistical mechanics to develop the first isotherm model for adsorption on an ideal flat surface assuming monolayer coverage [9]. Brunauer, Edward and Teller (BET) then expanded it to describe multilayer adsorption [10]. Empirical models have been proposed in addition to these theoretically derived isotherm models [11]. Every isotherm model has its range of applications, but most rely on assumptions, which are not necessarily fulfilled in real products or are limited to a specific product group. Another challenging part for the prediction of isotherms is the hysteresis effects. These are phenomena, by which a solid at a specific relative humidity and temperature has a lower water content during the adsorption process compared to the subsequent desorption process [12]. It has been shown that this is not only due to time dependencies, but this hysteresis appears also in steady-state [12]. One explanation for the hysteresis for porous solids is pore condensation and the inkbottle theory [13]. The inkbottle theory assumes that the pore network consists of a few large cavities connected with small and narrow pores (i.e., bottlenecks). There is free access to all parts of the pore network during the adsorption. However, during desorption the water condensed in the bigger pores is blocked from evaporating until all the smaller canals between the cavity and the surface are emptied, even if the pressure is already low enough that the water is thermodynamically unstable. Therefore pores should mainly affect the desorption process, while the adsorption process is relatively unaffected [13].

In summary, many different isotherm models already exist and are successfully used within their individual range of applications and boundaries. Therefore, this paper does not want to propose a new isotherm model. Instead, the experimentally generated data is used to calculate individual points on that isotherm for the different relative humidity and interpolates between this grid points. However, the isotherm only describes the equilibrium but not the kinetics, which are necessary for the integration into a complete shelf-life model, with varying storage conditions and the coupling of additional processes, like permeation through the packaging. Such kinetic behaviour is also important for the design of active packaging systems, like oxygen scavengers, antimicrobials and moisture absorbers, because multiple concurring processes occur simultaneously. In this study, a simple kinetic model for the sorption process is presented. Silica gel is chosen as a well-controllable system for general model development here instead of an actual food. Its main benefit is that it is chemically inert and stable during the experiment, which lasts for around three weeks. Actual food could decay during this time and distort the results. While silica may not be a quantitative proxy for a food system, the results on silica itself may still be quantitatively useful as silica gel is the most commonly used desiccant for preventing excess water in food packages [14] and is used as a humidity regulator in various applications like pharmaceuticals, artworks or electronics [15]. Reports regarding the sorption of water on different grades of silica gel already exist [15,16,17]. Nevertheless, previous research mainly focuses on the sorption isotherm, the behaviour at different temperatures or the calculation of the maximum capacity when used as a regulator to protect packed products. However, there is no systematic study of the kinetic effects of de-/sorption on silica gel.

## 2. Materials and Methods

### 2.1. Material

Silica gel beads with a grain size of 2–5 mm were obtained by Carl Roth, Karlsruhe Germany. Moreover, silica gel is a chemically inert, nontoxic material with a high internal surface area of around 750 m^2^/g. The pore volume of silica gel is around 0.4 cm^3^/g.

### 2.2. Sorption Measurement

The water vapour sorption measurements were carried out using the dynamic vapour sorption (DVS) system (“Model resolution”, Surface Measurement Systems, London, UK). Silica gel beads with a weight of approximately 0.05 g are placed inside a sealed chamber. During the experiment, the samples are exposed to a predefined relative humidity and temperature profile and are weighed every minute. Equilibrium is assumed, whenever the change in the measured weight over five consecutive data points is lower than a defined threshold. If all samples are in an equilibrium state, the experiment continues with the next step of the humidity profile.

**Figure 1:**
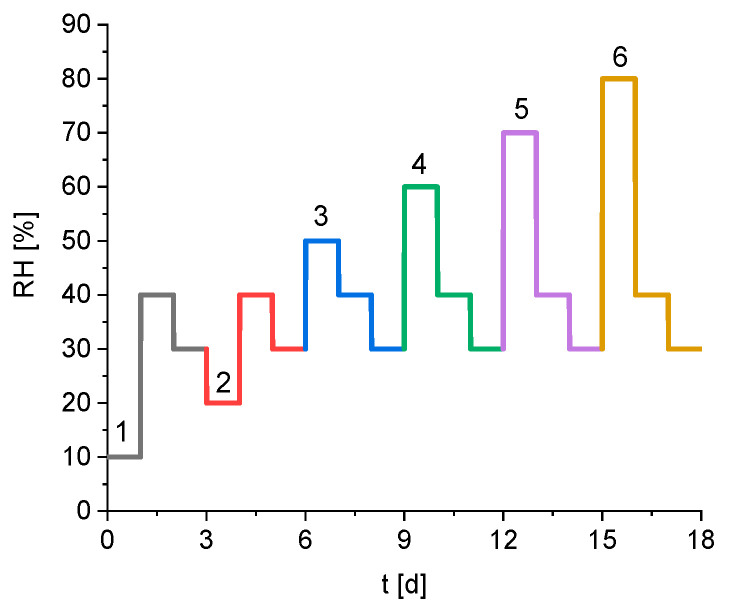
Predetermined humidity profile of the sorption experiment, the numbers mark the six different subparts during the experiment.

In this study, the relative humidity profile shown in Figure 1 was applied. The relative humidity of each profile step was kept constant for 24 h, even if the equilibrium was reached. Typically, the sorption behaviour of solids is measured by changing the relative humidity from 0% to 90% in constant steps of 10% [18,19]. Afterwards the relative humidity is reduced back to 0%, again in steps of 10%. This study uses a different approach to examine the effect of varying subsequent humidity levels. Firstly, it focuses on the region between 30% and 80% relative humidity because it is the primary area of hysteresis effects on the silica gel [20]. Different step heights in the relative humidity are used to characterise the kinetics depending on the deviation from the equilibrium. Alternating the increase and decrease of the relative humidity during the experiment also allows investigation of the effect of partial hysteresis on the kinetics. The final parameter that might influence the sorption behaviour is the relative humidity earlier in the process. Therefore, the applied relative humidity profile contains six steps, where the desorption of water after a change in the relative humidity from 40% to 30% is measured. The only difference is the relative humidity directly before that step, which is varied between 10% and 80% relative humidity and numerated from 1 to 6 in Figure 1. The temperature during the measurement is kept constant at 23 °C during the whole experiment.

### 2.3. Modelling of the Sorption Process

A pseudo-first order reaction (PFO) is a commonly used kinetic model to describe the adsorption kinetics for a wide variety of substances [21].

(1) $$\frac{dw}{dt}=k(f\left(RH\left(t\right)\right)\;\cdot \;\left(w_{iso}\left(f\left(RH\left(t\right)\right)\right)-w\left(t\right)\right)$$

In (1) $w_{iso}$ represents the equilibrium water content, which will be adsorbed onto the solid at the current relative humidity after infinite time. However, because of hysteresis effects$\;w_{iso}$ not only depends on the current relative humidity but can also depend on the exposure history to relative humidity earlier in the process. In (1) *k* is the reaction rate constant, which describes how fast the equilibrium will be reached. This parameter also depends on the current relative humidity and again can be affected by the history of relative humidity, because there are different sorption processes, like surface sorption, pore sorption and pore condensation occurring for different levels of relative humidity.

However, using the assumption that the equilibrium and the reaction rate are constant as long as the current relative humidity stays constant, the experiment shown in Section 2.2 can be divided into the 18 independent time steps $t_{i}$, each step having a duration of 24 h. Therefore, one ODE can be written for each step *I* of the experiment as:

(2) $$\frac{dw_{i}}{dt}=k_{i}\;\cdot \;\left(w_{i,iso}-w_{i}\left(t_{i}\right)\right)$$

Hereby $t_{i}$ represents the time since the start of the *i*-th step of the experiment. It has to be emphasized that the calculated reaction rate constant $k_{i}$ and the calculated isothermal water content $w_{i,iso}$ are only usable for the exact relative humidity profile of the experiment.

### 2.4. Modelling of the Sorption Experiment

During each individual step *i* of the humidity profile of the sorption experiment (Figure 1) the relative humidity in the head space is kept constant. The equilibrium water content $w_{i,iso}$ is the water content the sample reaches at this relative humidity after infinite time. Therefore, as long as the relative humidity is constant, $w_{i,iso}$ will also be constant.

The first-order differential Equation (2) can be solved analytically using the initial condition (3):

(3) $$w_{i}\left(t_{i}=0\right)=I$$

Hereby $w_{i,0}$ is the experimentally measured value at the beginning of the *i*-th step. The analytically solution for the water content is:

(4) $$w_{i}\left(t_{i}\right)=\left(w_{i,0}-w_{i,iso}\right)e^{-k_{i}t}+w_{i,iso}$$

It has to be emphasized that the pseudo-first-order approach for the description of the adsorption and desorption is not based on a physical explanation. Instead it uses a simplifying assumption commonly applied in literature because of its good agreement with experimental data for both the adsorption as well as the desorption [22].

The model parameters $k_{i}$ and $w_{i,iso}$ were fitted for each step of the RH profile using experimental data and the Matlab built-in least-squares solver *lsqnonlin* resulting in 18 reaction rate constants and isothermal water contents. *Lsqnonlin* uses the trust-region-reflective algorithm based on the Newton method described in [23]. The solver searches for a way to minimise the squared sums SSQ of the relative deviation of the predicted and the measured water content [24].

(5) $$SSQ_{i}=\overset{N}{\underset{j=1}{\sum }}{\left(\frac{w_{i,model,j}-w_{i.exp,j}}{w_{i.model,j}}\right)}^{2}$$

where *N* is the number of experimental observations per step *i*. Because each step has the same duration of 24 h and there is one measurement point per minute *N* has the same value for every step and can be calculated as 1440. The index *j* describes the 1440 experimental data points per step *i*. The quality of the fit is described using a relative root mean square error (6):

(6) $$RRMSE_{i}=\sqrt{\frac{SSQ_{i}}{N-p}}$$

where p is the number of fitted parameters. In this case, the number of fitted parameter per step is two; the reaction rate $k_{i}$ and the isothermal point$\;w_{i,iso}$. It has to be clarified that using this method the calculated equilibrium water content $w_{i,iso}$ does not necessarily equal the water content at the end of the measurement, if the system is not in the equilibrium at the end of the measurement. Instead, it is a parameter, which is estimated using the least square estimator and the experimental data and describes the calculated equilibrium water content after infinite time. This approach depends on the quality (signal/noise ratio) of the experimental data. Especially very small slopes that only show up at very long measurement times might be underestimated. Additionally, this extrapolation allows the measurement to be stopped before reaching equilibrium as long as the exponential characteristics suggested by the model (5) can be justified. This can lead to significant timesaving, especially for samples with overall slow kinetics. By comparison of $w_{i,iso}$ with the last experimental data point for a given RH it can additionally be checked in hindsight how close the process has actually approached equilibrium.

### 2.5. Hysteresis Effects in Porous Materials

The maximum pore radius r, when capillary condensation occurs at a given relative humidity can be calculated using the Kelvin Equation (8). The critical radius r means that all pores with a smaller diameter are filled with the liquid, and all pores with a bigger radius are filled with the gas [25]:

(7) $$\ln\left(\frac{p}{p_{s}}\right)=\ln\left(RH\right)=-\frac{2\gamma \nu }{rRT}$$

(8) $$r=-\frac{2\gamma \nu }{RT\;\ln\left(RH\right)}$$

where the molar volume is ν, the surface tension of water is γ, the gas constant is R, the saturation pressure of the water at the current temperature is $p_{s}$, and the current partial pressure of the liquid in the gaseous phase is *p*. Since the temperature during the experiment is kept constant, the surface tension, and the molar volume of the water are also constant. If the pore size distribution of a substance is known and it is assumed that the diameter of a pore stays constants over the complete pore length this would allow the calculation of a sorption isotherm. However, for the same relative humidity, Equation (8) always leads to the same critical pore diameter, and therefore the same water content, regardless of adsorption or desorption, and therefore cannot explain the hysteresis. Nevertheless, the pore network of the silica gel can explain this behaviour, because in reality, the pores of the silica gel are non-uniform and there are parts of the pore with a larger diameter, the cavities and parts with a narrower diameter, the bottlenecks. According to [13], when there is only a small amount of adsorbate there is free access to all parts of the internal volume, leading to a filling of the pores with increasing pore size. However, during the desorption, the small bottlenecks are filled with water and are blocking the evaporation of the water of the bigger cavities. Only if the relative humidity is so far reduced that the smallest diameter of the pore is below the critical diameter will the complete pore be emptied.

## 3. Results

An essential factor for shelf-life modelling is the water content of the product. Its behaviour is influenced by the sorption isotherm of the product and the sorption kinetics. Therefore, this study researched the influence of relative humidity on the sorption kinetics of silica gel as a model substance.

**Figure 2:**
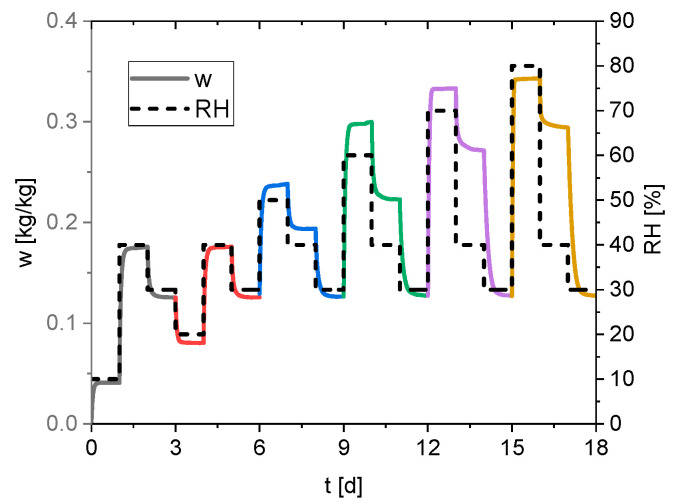
Measured water content of the silica gel (continuous line) and the set relative humidity profile (dashed line).

Figure 2 presents the experimental data from the sorption measurement. The relative humidity is kept constant for 24 h, and the water content of the solid phase is measured every minute.

The measured data is divided into 18 subsets for further analysis, one for each new relative humidity condition. The 18 subsets are then regrouped into 6 parts, each containing 3 subsets. Only the relative humidity of the first subset of each group is different and varies between 10% and 80% relative humidity. The second subset of each group has always a relative humidity of 40%, the third subset of each group has always a relative humidity of 30%. The fitted parameters $k_{i}$ and $w_{i,iso}$ for every subset are listed in Table 1. As comparison *w_i,final,mess_*_,_ which is the last measurement point of each subset is also included in Table 1.

**Figure 3:**
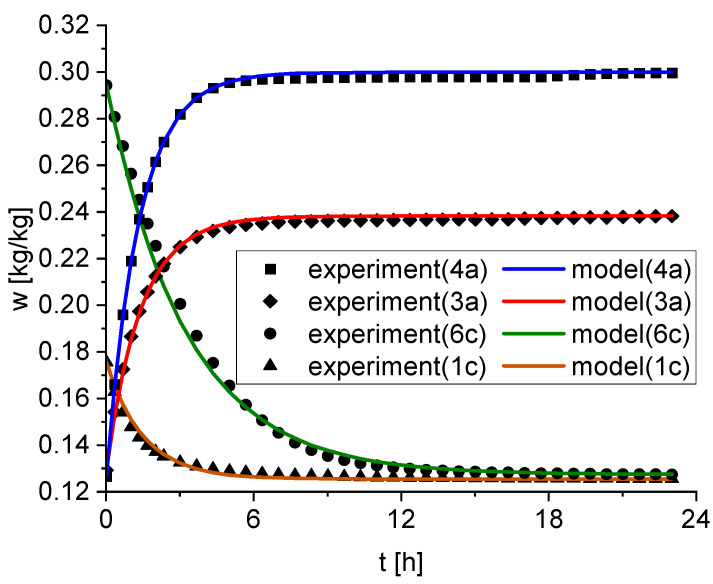
Comparison of the experiment and the model for four representative subsets (4a, 3a, 6c, 1a). The solid lines correspond to the model and the symbols are the experiment.

The reaction rate constants$\;k_{i}$ and the isothermal point $w_{i,iso}$ are determined by fitting the model described in Section 2.3 to the experimental data for each subset. Hereby, as mentioned in Section 2.4, does $w_{i,iso}$ not represent the final measurement point of each step after 24 h but the extrapolated equilibrium water content after infinite time. This allows a good estimation of the equilibrium water content even if the equilibrium between adsorption and desorption was not reached within the 24 h. However, a comparison of *w_i,iso_* and *w_i,final_._mess_* indicates that equilibrium was reached in this experiment for every step. The *RRMSE* calculates the average relative deviation between the experimental and predicted model values. For every subset, the deviation is less than two percent and demonstrates a good fit of the proposed model for the experimental data. As an example, Figure 3 shows the experimental data at four different conditions as well as the fitted model.

## 4. Discussion

### 4.1. Equilibrium Water Content of Silica Gel

**Figure 4:**
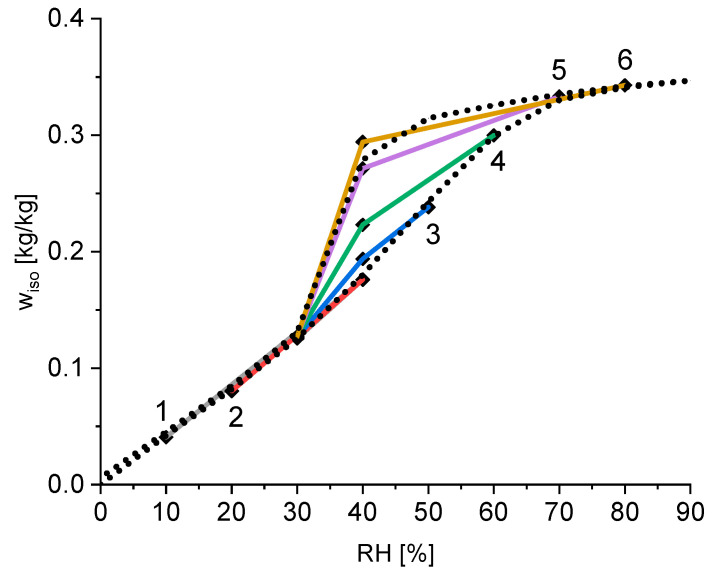
Calculated equilibrium water content of the silica gel for each subset of the experiment (points) and the measured reference isotherm (dotted line); each coloured lines connects the point of one subgroup.

The calculated equilibrium water content for every subset is shown in Figure 4. The equilibrium water contents of the three subsets in each group are connected to show the time course of the measurement. The first point of each group is highlighted with the group number from 1–6. The final point of each group is for a relative humidity of 30%. This water content is almost identical for every group (see also Table 1) and therefore only represented as one point for all groups. For the better evaluation of these results, Figure 4 also shows the measured isotherm of this silica gel using the standard procedure of starting the measurement at 0% RH, followed by an increase of 10% each time, until 90% RH is reached and measuring the equilibrium water content each time. Afterwards, the RH is reduced to 0% RH, again in steps of 10%.

The measurement of the standard isotherm shows that the hysteresis effects of silica gel are located in the relative humidity range between 30% and 80%. It can be seen that there is a linear correlation of the water content and the relative humidity for a relative humidity up to 30%. As seen in Table 1 the isotherm water content for a relative humidity of 30% is around 0.13 kg/kg independent of the sample history. These two observations suggest that for a relative humidity of 30% or lower, the adsorption occurs only on the outside area of the silica particles and the thickness of the outside water layer is proportional to the partial water pressure. Figure 4 also shows that there is no hysteresis for low relative humidity below 30%, because the surface adsorption on the outside is a reversible process. However, for higher relative humidity, pore condensation occurs. Especially interesting are the different water contents at a relative humidity of 40%. Hereby, a higher former relative humidity leads to higher equilibrium water content at the same relative humidity of 40%. This is again a validation of the ink-bottle theory explained in Section 2.5. A higher relative humidity in the previous step leads to the filling of bigger cavities inside the silica gel and therefore a higher water content. The followed reduction to 40% RH reduces the critical pore diameter and all pores, where the smallest bottleneck is bigger than the critical diameter will empty. The pores with a smaller bottleneck remain filled. The amount of water remaining depends on the amount of water adsorbed at the previous step, which correlates with the relative humidity at that step. Therefore, as seen in Figure 4, a higher relative humidity at the previous step leads to a higher water content at 40% RH. Only after reduction to 30% RH is the partial pressure of water reduced enough that almost all pores are emptied, and the hysteresis vanishes.

This finding also indicates that there are not only two states for the isotherm, one for the adsorption and one for the desorption. Instead, there are multiple states with a partial hysteresis depending on the history of the sample.

### 4.2. Sorption Kinetics of Silica Gel

**Figure 5:**
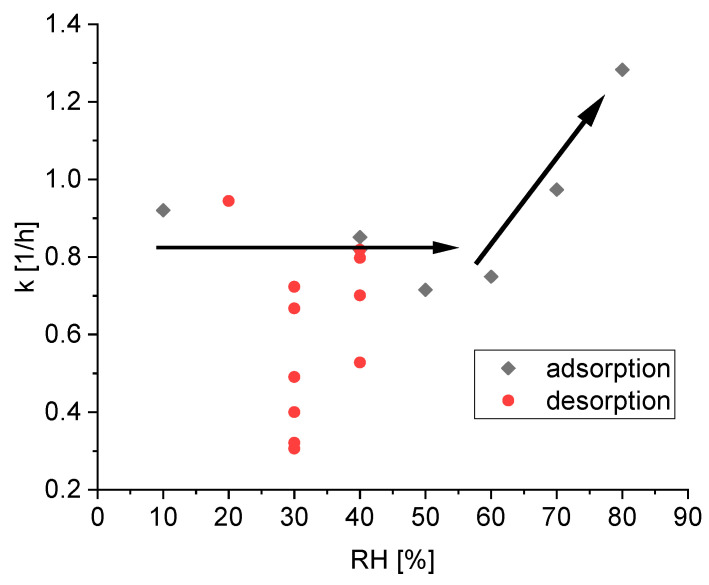
Calculated reaction rate constants k during the adsorption (black points) and desorption (red points) process of water on silica gel; the black arrows are added to visualize the tendency of the reaction rate constant of the adsorption.

Figure 5 presents the fitted reaction rate constants *k* of silica for all 18 subsets.

The data for the kinetic of the adsorption is not quantitatively fully conclusive. However, the apparent trends can be physically interpreted. For low relative humidity below 60%, the reaction rate k seems independent from the relative humidity and the scatter may be attributed to the model simplifications and slight experimental errors. The main factor of water uptake in silica gel between 30% relative humidity and 80% relative humidity is due to pore condensation. Condensation in pores with higher diameter is possible with higher relative humidity and therefore higher water pressure in the gaseous phase. Thus, the independence of the calculated reaction rate constant *k* of the relative humidity suggests that the kinetics of the pore condensation is independent of the pore diameter. This finding also validates the ink-bottle theory, which suggests only influences on the desorption process, not on the adsorption process [13]. Only for high relative humidity above 70%, can a speedup in the kinetics be observed. At this point, the pores are filled and the condensation on the outside surface again is the main factor.

Figure 5 also shows the fitted reaction rate constants k of silica for all 11 subsets, where silica gel releases water, i.e., the current relative humidity is lower than the relative humidity in the previous step. A comparison of the adsorption and desorption kinetics indicates an overall slower sorption rate constant for the desorption compared to adsorption. However, there is a higher fluctuation between the different values. While some kinetic constants for the desorption are in the same range as the kinetic constants for the adsorption the lowest kinetic constant is five times slower. However, there is no clear relationship between the reaction rate and the current relative humidity.

**Figure 6:**
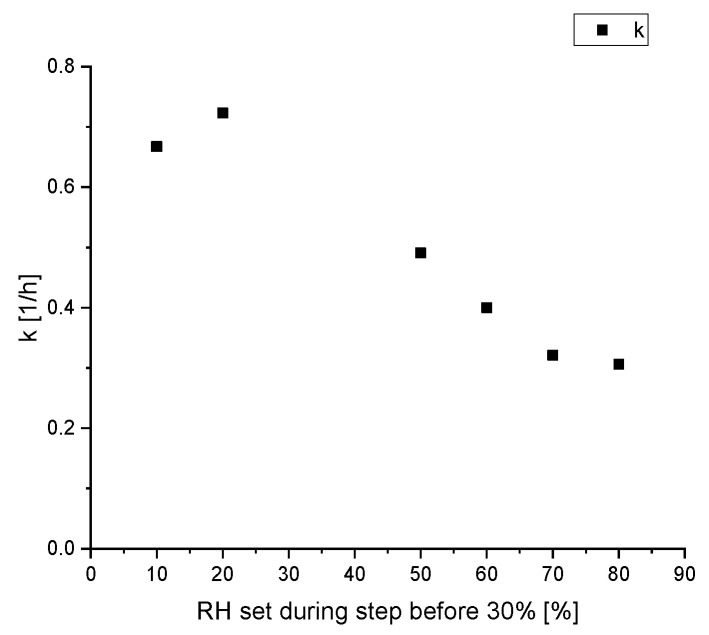
Calculated reaction rate constant *k* during the desorption process of water on silica gel at a relative humidity of 30%, depended on the former relative humidity.

The different values at a relative humidity of 30% are noteworthy, suggesting the values of the reaction rate constant k does not only depends on the current relative humidity. A closer look at this phenomenon is given in Figure 6. There are six subsets, where the desorption kinetic is measured at a relative humidity of 30% after being at 40%. The only difference is the relative humidity RH_history_ two steps before, which is varied between 10% and 80%.

Figure 6 shows a decreasing reaction rate constant k with a higher former relative humidity. While the desorption rate constant after a low former relative humidity of 10% and 20% relative humidity is comparable to the rate during the adsorption, the value significantly decreases with a former relative humidity of 50% and higher. These results suggest that the desorption kinetics of water on silica gel depends not only on the current relative humidity but also the pathway of the relative humidity, the silica gel was exposed to. This is another consequence of the hysteresis effect. Below 40% relative humidity there is no significant hysteresis because the sorption on the outer surface of the silica gel particles is the main factor, similar to the Langmuir isotherm. This is a simple reversible process and with a decrease in relative humidity, the water desorbs with the same kinetics as it adsorbed. Therefore, the reaction rate constants for adsorption and desorption are similar, explaining the comparable values for the kinetic constants with a RH_history_ of 10% and 20% in Figure 6. For higher relative humidity, the influence of pore condensation surpasses the surface adsorption because of the higher internal surface area of silica gel compared to the outside surface area. According to inkbottle theory, after being exposed to high relative humidity the desorption process starts with the bigger pores at the surface, which are not blocked by smaller pores. Only once the partial pressure of water is below the capillary pressure of the smallest part of the pore can the water behind the bottleneck evaporate. Therefore, in this experiment the distribution of the remaining water in the silica gel particles at a relative humidity of 40% is different depending on the former relative humidity. After exposure to 70% relative humidity, followed by 40% relative humidity more pores with a larger diameter can still contain condensed water than the pores, which were only exposed to a maximum relative humidity of 50%. After another reduction of the relative humidity to 30% the vapour pressure, even in the smallest pores is low enough, such that the remaining water can evaporate. These smaller pores now limit the overall release kinetics of the remaining water. Therefore, this simplified model’s fitted reaction rate constant is lower if the silica gel was exposed to higher relative humidity, and therefore contains more remaining water. After the desorption at a relative humidity of 30%, the pores are entirely empty and the remaining water content is only at the outer surface of the silica gel, which is independent of the history of the silica gel. Therefore, the history of the relative humidity profile is only relevant for humidity above 30%.

## 5. Conclusions

It could be shown that the desorption kinetic of water on silica gel depends not only on the current relative humidity, but also on the relative humidity earlier in the process, because of hysteresis effects. A closer look at scatter on the fitted reaction constants shows a difference between adsorption and desorption. While both were not constant for individually fitted parts of the experimental data, the scatter was lower for the absorption process. In both cases, the experimental data can be described using an exponential expression containing only one free parameter, the reaction rate constant *k*. It is also shown that this exponential behaviour can be derived mathematically using two first order reactions for the evaporation and condensation of water onto the silica gel. These results can also help by the design of more efficient packaging concepts for preservation of food or artwork. It shows that more silica gel is needed to protect against drying compared to moisturizing of the product because of the slower desorption kinetics of silica gel. The knowledge of the partial hysteresis of silica gel also allows the more detailed calculation of the water uptake or release capacity of silica gel under varying outside relative humidity, also helping by the design of the active packaging. Additionally, these experimental results can be used as a proof-of-concept for this measurement setup with a varying relative humidity profile, and can be used on perishable food in the future.

## Figures and Tables

**Table 1:** Fitted model parameter $k_{i}$ and $w_{i,iso}$ for the 18 subsets; measured parameter$\;w_{i,final,mess}$ and the *RRMSE* of each subset.

Subset	RH	$k_{i}\;[1/h]$	*w_i,iso_* [kg/kg]	*w_i,final_._mess_* [kg/kg]	$RRMSE_{i}\;[\%]$
1 a	10	0.920	0.041	0.041	1.81
1 b	40	0.851	0.176	0.176	0.94
1 c	30	0.667	0.126	0.125	0.81
2 a	20	0.944	0.080	0.080	0.27
2 b	40	0.819	0.176	0.176	0.50
2 c	30	0.723	0.126	0.126	0.66
3 a	50	0.715	0.238	0.238	0.60
3 b	40	0.818	0.194	0.194	0.28
3 c	30	0.491	0.127	0.126	0.70
4 a	60	0.749	0.300	0.300	0.48
4 b	40	0.701	0.223	0.223	0.87
4 c	30	0.400	0.127	0.127	0.58
5 a	70	0.976	0.333	0.333	0.74
5 b	40	0.528	0.272	0.271	1.31
5 c	30	0.321	0.127	0.127	0.73
6 a	80	1.283	0.343	0.343	1.10
6 b	40	0.798	0.294	0.294	1.10
6 c	30	0.306	0.127	0.127	1.59

## Data Availability

Not applicable.
